# Determinants of Extraocular Muscle Volume in Patients with Graves' Disease

**DOI:** 10.1155/2012/368536

**Published:** 2012-02-15

**Authors:** Samer El-Kaissi, Jack R. Wall

**Affiliations:** ^1^Specialized Diabetes and Endocrine Centre, King Fahad Medical City, Dabab Street, P.O. Box 59046, Riyadh 11525, Saudi Arabia; ^2^Department of Medicine, Nepean Clinical School, Nepean Hospital, The University of Sydney, Derby Street, P.O. Box 63, Penrith 2751, New South Wales, Australia

## Abstract

*Background*. To examine factors contributing to extraocular muscle (EOM) volume enlargement in patients with Graves' hyperthyroidism. *Methods*. EOM volumes were measured with orbital magnetic resonance imaging (MRI) in 39 patients with recently diagnosed Graves' disease, and compared to EOM volumes of 13 normal volunteers. Thyroid function tests, uptake on thyroid scintigraphy, anti-TSH-receptor antibody positivity and other parameters were then evaluated in patients with EOM enlargement. *Results*. 31/39 patients had one or more enlarged EOM, of whom only 2 patients had clinical EOM dysfunction. Compared to Graves' disease patients with normal EOM volumes, those with EOM enlargement had significantly higher mean serum TSH (0.020 ± 0.005 versus 0.007 ± 0.002 mIU/L; *P* value 0.012), free-T4 (52.9 ± 3.3 versus 41.2 ± 1.7 pmol/L; *P* value 0.003) and technetium uptake on thyroid scintigraphy (13.51 ± 1.7% versus 8.55 ± 1.6%; *P* value 0.045). There were no differences between the 2 groups in anti-TSH-receptor antibody positivity, the proportion of males, tobacco smokers, or those with active ophthalmopathy. *Conclusions*. Patients with recently diagnosed Graves' disease and EOM volume enlargement have higher serum TSH and more severe hyperthyroidism than patients with normal EOM volumes, with no difference in anti-TSH-receptor antibody positivity between the two groups.

## 1. Introduction

Thyroid-associated ophthalmopathy (TAO) is an autoimmune disorder of uncertain aetiology. While the involvement of extraocular muscles (EOMs) in patients with Graves' disease may seem infrequent on clinical examination, orbital magnetic resonance imaging (MRI) studies suggest that the majority of such patients have EOM enlargement [1]. 

Along with the orbital fibroblast, the EOM is likely to be a primary target in TAO. This is supported by evidence of T-cell reactivity against both orbital fibroblast and EOM cells *in vitro *[2] and muscle fibre damage in electron microscopic studies of EOM from patients with recent onset TAO [3, 4]. In addition, expression of the thyrotropin-receptor (TSH-R) in EOM [5, 6], as opposed to the widespread distribution of TSH-R in adipose tissues throughout the body [7], may indicate that EOMs have a more direct and specific role in TAO than previously thought [8].

In this study, we investigated potential factors affecting EOM volume enlargement as measured by orbital MRI in patients with recently diagnosed Graves' disease.

## 2. Materials and Methods

A total of 39 patients diagnosed with Graves' hyperthyroidism within the preceding 3 months were selected for this study. The patients were involved in a larger study looking at potential risk factors for TAO [9].

The diagnosis of Graves' disease was based on the presence of biochemical hyperthyroidism, a symmetrical goitre and positive thyroid autoantibodies, and/or diffuse uptake on ^99m^Technetium thyroid nuclear scan. The abbreviated clinical activity score (CAS) model was employed for the diagnosis of active ophthalmopathy. This model assigns one point for each of the following: spontaneous retrobulbar pain, pain on eye movement, eyelid erythema, eyelid oedema, chemosis, conjunctival injection, and swelling of the caruncle [10]. A total score ≥4 out of 7 was defined as active ophthalmopathy [11]. EOM function was evaluated by asking the patient to move their eyes in an H-shaped pattern, and proptosis was assessed using a Hertel exophthalmometer. The ophthalmic examination was performed by a trained clinical nurse.

Patients who were pregnant, less than 18 years of age, and those with a history of radioactive iodine therapy, orbital surgery, orbital irradiation, or significant loss of vision were excluded. The study was conducted at an outpatient endocrine practice in Victoria, Australia. Written, informed consent was obtained, and the study was approved by the Barwon Health Research and Ethics Advisory Committee.

EOM volumes were measured by a single investigator (SEK) from T_1_-weighted, 2 mm slice orbital MRI scans using the digital software MRIcro (Version 1.38 Beta; Chris Rorden) as previously described [9]. Briefly, the volumes of the medial, inferior, and lateral recti were measured manually by circling the muscle perimeter on each slice. The superior rectus muscle, the superior ophthalmic vein, and the levator palpebrae superioris were measured together as the superior muscle group (SMG) because of difficulties in delineating these structures from each other. Orbital measurements were expressed as a percentage of the mean globe volume for each patient in order to adjust for interindividual variation in EOM volumes.

## 3. Statistical Analysis

Statistical analysis was performed with the software programs Minitab 14.12 and SPSS 13.0. Proportions were compared with Fisher's exact test while the sample means were evaluated with the 2-sample *t*-test. The effect of TSH-R antibody positivity on EOM volume was examined with binary logistic regression. Significance was set at *P* value less than 0.05.

The cutoff values of MRI-measured EOM volumes were determined with receiver-operating-characteristic analysis by comparing patient EOM volumes to those of 13 normal volunteers with no history of thyroid or eye disease. The cutoff values, their sensitivities and specificities, and the coefficients of variation of each measurement were detailed in an earlier publication [9].

## 4. Results

Based on the EOM volume cutoff value, only 8 patients had normal volumes in all 4 EOM groups. Of the 31/39 patients with at least one enlarged EOM volume, 3 patients had one enlarged EOM, 3 patients had 2 enlarged EOM, 7 patients had 3 enlarged EOM, and all 4 muscles were enlarged in 18 patients. The most frequently affected EOMs were the medial and lateral (*n* = 27 each) followed by the inferior recti and SMG (*n* = 24 each).

Assessment of baseline characteristics in patients with and without EOM volume enlargement on MRI showed no significant differences in the proportion of males, tobacco smokers, those with active ophthalmopathy (CAS ≥ 4), or elevations in anti-TSH-R, antithyroid peroxidase (TPO), or antithyroglobulin autoantibodies (Table 1). Only two patients, both with EOM volume enlargement, had clinically evident EOM dysfunction.

However, patients with enlarged EOM volumes had significantly higher ^99m^technetium uptake on thyroid scintigraphy and greater serum free-T4 and thyrotropin (TSH) levels (Table 2). Importantly, there were no significant differences between the two groups in the proportion of patients who received anti-thyroid medications prior to recruitment into the study. Overall, 8/31 patients with enlarged and 2/8 patients with normal EOM volumes received anti-thyroid medications for a mean 1.06 ± 0.45 weeks and 1.13 ± 0.79 weeks, respectively. Exclusion of those patients from the analysis resulted in persistent elevations of free-T4 and TSH levels in patients with EOM enlargement, although the differences in mean TSH became of borderline significance (mean free-T4 42.4 ± 1.9 versus 52.3 ± 3.9 pmol/L (*P* value 0.034); mean TSH 0.021 ± 0.006 versus 0.008 ± 0.003 mIU/L (*P* value 0.054)).

In further analysis using binary logistic regression, EOM volumes were not associated with elevated anti-TSH-R, anti-TPO, or antithyroglobulin autoantibodies, smoking, or the presence of active ophthalmopathy.

## 5. Discussion

This study shows that patients with newly diagnosed Graves' disease and EOM enlargement have higher serum TSH and more severe hyperthyroidism, as suggested by the higher serum free-T4 and greater uptake on thyroid scintigraphy, than patients without EOM enlargement.

While more severe hyperthyroidism has not been identified as an independent risk factor for TAO [12], a greater serum free-T3 at baseline is associated with an increased risk of TAO after radioiodine therapy for Graves' hyperthyroidism [13]. In this study, EOM volume enlargement was associated with higher free-T4 levels (*P* value 0.003; Table 2) and greater uptake on thyroid scintigraphy (*P* value 0.045; Table 2). The serum free-T3 level was greater in patients with EOM volume enlargement without reaching statistical significance (*P* value 0.062; Table 2), perhaps due to the small sample size. The mechanism whereby more severe hyperthyroidism leads to greater EOM volumes is uncertain, but we speculate that it may be related to higher levels of the shared thyroid-orbital antigen(s).

In this study, the mean serum TSH was significantly higher in patients with EOM volume enlargement. The role of serum TSH in the initiation and propagation of TAO is well documented after RAI therapy [13–16], and empirical thyroid hormone replacement after RAI ablation, but before the onset of biochemical hypothyroidism, has been shown to reduce the incidence of TAO after RAI [17]. It is possible that EOMs, which express TSH-R [5, 6], are sensitive to seemingly minor elevations in serum TSH in patients with Graves' hyperthyroidism, leading to greater EOM volumes. The higher TSH levels in patients with enlarged EOM volumes occurred despite higher free-T4 and free-T3 levels in this group. While the serum TSH usually changes in a reciprocal fashion to the serum free-T4 and free-T3 levels, it is worth noting that this relationship is attenuated or “flattened” in hyperthyroid patients with a suppressed serum TSH below 0.01 mIU/L [18]. Upon starting treatment with anti-thyroid medications, the serum free-T4 and free-T3 fall rapidly whereas the serum TSH typically “lags” behind and remains undetectable for up to 3 months [18]. It is therefore unlikely that the greater serum TSH in patients with EOM volume enlargement was related to treatment with anti-thyroid medications prior to recruitment into the study, especially because the mean duration of treatment with anti-thyroid medications was 1.08 ± 0.39 weeks, and did not exceed 3 months in any patient. In addition, exclusion of patients who received anti-thyroid medications from the analysis did not abolish the differences in serum TSH between the two groups, although the differences became of borderline significance (*P* value 0.054), possibly due to the smaller sample size.

While the majority of patients in this study had elevated anti-TSH-R antibody levels, there were no significant differences in the prevalence of anti-TSH-R antibody positivity between patients with and without EOM volume enlargement, and in binary logistic regression analysis there was no association between antibody positivity and EOM volumes. TAO is thought to occur following sensitization of T-lymphocytes to a common thyroid and orbital antigen. The identity and location of this antigen remains unknown, but the TSH-R is the most likely candidate [7, 19]. Autoimmunity against other antigens particularly the skeletal muscle protein calsequestrin [20] is of potential importance, but is not well understood. The role of TSH-R in the initiation and propagation of TAO is supported by the close temporal relationship between the onset of ophthalmopathy and Graves' disease which is caused by stimulating anti-TSH-R antibodies [12], and the positive correlation between these antibodies and the prevalence of TAO in untreated Graves' disease [21]. In addition, TSH-R antibody levels are closely associated with CAS readings, the severity of the eye disease [22], and to a lesser extent with proptosis [23]. Therefore, the lack of an association between TSH-R antibody positivity and EOM volume enlargement in this study should be interpreted with caution, especially because of the small sample size and the increased risk of a type 2 error.

Similarly, the small sample size may account for nonsignificant differences in measures of proptosis, which was greater in patients with enlarged EOM volumes without reaching statistical significance (*P* value 0.097; Table 2). In contrast, the lack of an association between active ophthalmopathy and EOM enlargement may be related to the use of the CAS model which measures soft tissue and periorbital inflammation rather than EOM involvement [10, 24].

## 6. Conclusions

In patients with newly diagnosed Graves' disease, EOM volume enlargement is associated with greater serum TSH levels and more severe hyperthyroidism, as suggested by greater serum free-T4 levels and more avid uptake on thyroid scintigraphy. There was no association between EOM volumes and anti-TSH-R antibody positivity, although the small sample size may have contributed to this negative finding. Larger studies are needed to examine the relationship between serum TSH, anti-TSH-R antibodies, and EOM enlargement.

##  Conflict of Interests 

The authors declare that there is no conflict of interests.

## Figures and Tables

**Table 1:** Comparison between patients with and without EOM volume enlargement. The number of patients is shown, and the proportions *P* value was calculated using Fisher's exact test.

	Enlarged EOM	Normal EOM	*P* value
	volume (*n* = 31)	volume (*n* = 8)	
Males	3	1	1.0
Smokers	9	3	0.7
Active ophthalmopathy	15	3	0.7
Clinical EOM dysfunction	2	0	1.0
Elevated TSH-R antibodies	23	7	0.6
Elevated thyroglobulin antibodies	14	5	0.4
Elevated TPO antibodies	19	7	0.2

**Table 2:** Means ± SEM of measurements for patients with and without EOM volume enlargement. Where the measurement was not performed on all patients, the number of patients is shown in square brackets. *P* values calculated using 2-sample *t*-test.

	Enlarged EOM	Normal EOM	*P* value
	volume (*n* = 31)	volume (*n* = 8)	
Age (yrs)	44 ± 2.1	40.5 ± 4.1	0.5
^ 99m^Technetium uptake (%)	13.51 ± 1.7, (*n* = 28)	8.55 ± 1.6, (*n* = 6)	0.045
TSH (mIU/L)	0.020 ± 0.005	0.007 ± 0.002	0.012
Free-T4 (pmol/L)	52.9 ± 3.3	41.2 ± 1.7	0.003
Free-T3 (pmol/L)	22.8 ± 1.9, (*n* = 29)	18.3 ± 1.3	0.062
CAS	2.97 ± 0.53	3.25 ± 1.2	0.8
Proptosis (mm)	17.46 ± 0.46	16.00 ± 0.71	0.097
